# Consolidation of the Journal *Investigación y Educación en Enfermería* as a Publication with Impact on the Dissemination of Nursing Knowledge*

**DOI:** 10.17533/udea.iee.v41n3e01

**Published:** 2023-10-26

**Authors:** María de los Ángeles Rodríguez-Gázquez

**Affiliations:** 1 RN, Ph.D. Full Professor at Facultad de Enfermería de la Universidad de Antioquia, Colombia. Editor of the journal Investigación y Educación en Enfermería from 2010 to the present. Email: maria.rodriguezg@udea.edu.co Universidad de Antioquia Universidad de Antioquia Colombia maria.rodriguezg@udea.edu.co

Almost 14 years ago, Dean Beatriz Elena Ospina Rave entrusted me with the direction of the IEE Journal, given the leave of absence taken by the then director, María del Pilar Pastor, who had been named Secretary of Health of the Municipality of Medellín. Meeting this responsibility represented a great academic and professional challenge because it was a scientific publication of the highest quality in the area of Nursing, recognized in Colombia and Latin America. I received a journal with 26 years of uninterrupted edition and which already had three years in category A2 in the PUBLINDEX by COLCIENCIAS, hence, I had the commitment to lead the preparation of the publication to ascend to the long-awaited maximum A1 category, for which it was “only” necessary to meet the most complex criterion to obtain: achieve indexing in Medline, or in at least one of the bibliographic citational bases, like Elsevier's Scopus or in the Web of Science, now Clarivate.

The first thing I did in 2010, which I now do not recommend to any editor under any circumstance, was to send the journal for simultaneous evaluation in Medline, Scopus, and WoS. The result: the three databases rejected indexing, but nourished their assessments with invaluable recommendations, some of which were implemented with minor difficulty due to the high level the journal already had, such as increasing the periodicity from two to three issues per year and performing some minor adjustments in the editorial process. But other observations were profoundly debated in the Editorial Committee and in meetings with the Faculty's professors, given that they included substantive aspects; the most controversial was that of changing the publication language from Spanish to English to improve the dissemination of the articles so that they were accessible, by language, at a global level. Although some professors, at the time, predicted the journal’s demise if it were published in English, the truth is that since this requirement was approved in 2012 the journal began to receive a greater number of articles, a situation that has increased to avalanche level in recent years. 

Currently, eight of every ten articles come from outside Colombia, with significant contribution from Latin America and a large increase of manuscripts received from countries in Europe, Asia, and Oceania. In every article it is seen that the ways in which Nursing faces the challenge of providing care to people in any part of the world are common. In 2013, after having adjusted all the recommendations by the three bibliographic databases, we put together the corresponding packages to mail and gave these our blessings before they left the office. The result of this second evaluation was the approval for indexing in Medline in 2014 and in Scopus in 2015; in the latter, we were classified with an honorable quartile 3! With WoS, although not formally rejecting us, did write to us that “Nursing was not a priority area for indexing at that time”, a phrase that still fills me with indignation.

Due to the heated discussion about changes in the PUBLINDEX measurement model, between 2014 and 2016 no calls were made and, consequently, IEE retained the A2 category during those years, although already more than meeting the requirements for the expected A1. In 2017, the new PUBLINDEX model went into effect, much tougher than the prior, with the consequence that almost half of the Colombian scientific journals were eliminated from the system and the rest were downgraded. To reach the A1 category in the new model, the journal needed to be classified in quartile 1 by one of the two citational bibliographic bases; to maintain the A2 category it was essential to have reached quartile 2; so, because we were in quartile 3 in Scopus, we dropped from classification A2 to B, but that did not discourage us and we continued working for an editorial process of excellence.

Something quite important took place in 2019: committed to caring for the planet and abiding by the global recommendation to struggle against deforestation and contribute to reducing greenhouse gas emissions, the IEE Journal, after 36 years of being printed, became an exclusively electronic publication. The huge effort to print up to 1-thousand copies per number, the search for subscribers, the delivery and exchange of the physical journal were, with much difficulty, gradually reduced during the 10-year trajectory in which the printed and electronic versions coexisted, strengthening the latter with dissemination strategies of full-text articles and of free reading, that were available on the Internet sites of the many bibliographic bases in which we are indexed. In addition, the complete collection with articles since 1983 was updated on our site on the journal portal of Universidad de Antioquia.

Those high-profile bibliographic bases played an important role for *Investigación y Educación en Enfermería* to adapt to global demands for continuous improvement to align with the digital needs of the translation of what is the governance of the journal, which becomes visible when reviewing the editorial reach, practices, and procedures so that the articles can be read not only by humans, but by machines through metadata, which helped in the dissemination of the contents endorsed on our pages. Thus, in 2020, we were indexed by the most important bibliographic database in the world in health sciences: PubMed Central. This fact improved the journal's visibility by increasing the number of times our articles were cited, reflected in the change of classification from quartile 3 to 2 in Scopus since 2021. By the following year, IEE had another grand achievement: after four failed attempts, it was indexed in the world’s most recognized bibliographic citation base: Web of Science; in the Journal Citation Report we obtained an impact factor of 2, meaning that each article published in the journal is cited on average two times. This value also tells us that we are the nursing journal with the highest impact in Latin America.

And, now what? What does destiny have in store for us? Our obligation is to continue with the registration and observance of the criteria by indexing bases, for the maintenance and, hopefully improvement of the metrics as quality indicators of the journals. We also have great challenges to make the transition from Open Access to Open Science a reality. It is true that we have already made enormous strides: since 2009, we have full-text articles available, free of charge to publish or read; since 2017, all the articles have DOI identification; since 2020, authors’ ORCIDs are shown and the declaration of the use of the Creative Commons license is added that requires the attribution of authorship, non-commercial use and distribution under the same original license (BY-NC- SA). Moreover, we are indexed in the Directory of Open Access Journals (DOAJ), where we soon expect to obtain the golden seal. There are other challenges that need improvement, one is to achieve greater dissemination in social and academic networks. We must also advance in the mechanisms we will adopt to show the world transparency in all the stages of the editorial process: issues of open peer review, open data provision, and continuous publication should be discussed for possible incorporation. The future will dictate where to continue.

Lastly, I would like to thank the Faculty of Nursing for having allowed me to be the journal’s editor for almost 14 years, during which I have learned from the successes, but more from the mistakes made. I highlight the responsibility and academic judgment of the 11 professors who preceded me as head of the journal; without them, the achievements reached by the IEE would not have been possible; with them, I share 40 years of the journal’s uninterrupted publication. We have been responsible for the edition of 95 issues that already compile 1,173 articles, thus, promoting the fulfillment of the social purpose of legitimizing, storing, and keeping a rigorous record of the scientific knowledge of nursing to make it accessible in the global setting. Also, the authors who have written in our pages deserve very special thanks, as well as those individuals who are guarantors of the journal's quality and who support us in the internal and external Editorial Committees. We must also exalt the work by the experts who have participated in the irreplaceable evaluation of the articles, which has been an example of equanimity and independence of judgment. 

I also thank our esteemed monitors and those who have collaborated with us in the translation and revision of the texts with such rigor. Furthermore, it is necessary to recognize our readers who, during these 40 years, have accompanied us in this space for reflection on knowledge aimed at improving the care of people. We count on everyone's perseverance and enthusiasm to continue making this the best means of dissemination!

